# Utility of high-resolution anterior segment optical coherence tomography in the diagnosis and management of sub-clinical ocular surface squamous neoplasia

**DOI:** 10.1186/s40662-019-0152-3

**Published:** 2019-08-27

**Authors:** Ann Q. Tran, Nandini Venkateswaran, Anat Galor, Carol L. Karp

**Affiliations:** Department of Ophthalmology, Bascom Palmer Eye Institute, University of Miami Miller School of Medicine, 900 NW 17th St, Miami, FL 33136 USA

**Keywords:** Ocular surface squamous neoplasia, High-resolution optical coherence tomography, Sub-clinical ocular surface squamous neoplasia, Ocular surface imaging; ocular surface lesions

## Abstract

**Background:**

To evaluate the frequency and characteristics of sub-clinical ocular surface squamous neoplasia (OSSN) detected by high-resolution anterior segment tomography (HR- OCT) in patients with clinically unapparent disease following topical treatment.

**Methods:**

A retrospective chart review of patients with OSSN identified through a pharmacy database at the Bascom Palmer Eye Institute from January 2013 to December 2018 was conducted. Patients undergoing primary therapy with topical 5-fluorouracil 1% (5-FU) (4 times a day for 7 days with a 21-day break) or interferon-alpha-2b (IFN) (4 times a day) were reviewed. Patients were separated into two groups. Group 1 included individuals whose clinical resolution of OSSN aligned with complete resolution on HR-OCT. Group 2 (sub-clinical OSSN group) included individuals with clinical OSSN resolution but with features of persistent disease on HR- OCT. Patients excluded included those treated at an outside institution and those who used topical therapy as a surgical adjunct.

**Results:**

A total of 95 patients (95 eyes) were reviewed. Sub-clinical OSSN was detected at a frequency of 17% in our study patients (*n* = 16 patients, 9 treated with 5-FU and 7 treated with IFN). In the 16 individuals, the mean time to clinical resolution was 3.6 ± 1.0 cycles for 5-FU and 4.0 ± 0.0 months for IFN. An additional 2.1 ± 0.8 cycles for 5-FU and 1.2 ± 0.4 months for IFN were needed to achieve HR-OCT resolution of OSSN. Recurrence in Group 1 was noted in 10 patients (12%) while no recurrences occurred in Group 2, the cohort with subclinical disease that received the extended medical therapy. The mean follow-up was 24.0 ± 17.9 months.

**Conclusion:**

We found that at least 17% of individuals with apparent clinical resolution of OSSN have sub-clinical disease detected on HR-OCT. This information can be used to optimize treatment and extend therapy past the point of clinical resolution.

## Background

Ocular surface squamous neoplasia (OSSN) represents the most common epithelial squamous tumor of the ocular surface [1]. Surgical excision has been the traditional treatment for the disease. However, incomplete excision can result in microscopic disease with recurrences reported in up to 33–56% of cases [2]. The use of anterior segment high-resolution optical coherence tomography (HR-OCT) provides a non-invasive imaging modality to aid in the diagnosis and management of OSSN [3]. Classically, radiographic findings on HR-OCT include hyperreflective, thickened epithelium with an abrupt transition between normal and cancerous epithelium [4].

Topical chemotherapy has evolved as a common primary treatment for OSSN [5]. It has proved to be a highly effective treatment modality, with the most commonly used chemotherapeutic agents being interferon (IFN), 5-fluorouracil 1% (5-FU), and mitomycin C (MMC) [6]. These agents have high success rates and are used until the lesion is clinically resolved. Potential side effects of topical therapy include pain, hyperemia, conjunctivitis, and corneal toxicity, which are most prominent with MMC and least prominent with IFN [7].

HR-OCT provides a non-invasive imaging modality to aid in the diagnosis and management of OSSN [3]. The ability to obtain cross-sectional images of the ocular surface provides an “optical” biopsy in various corneal pathologies [8, 9]. The classic radiographic findings of OSSN help differentiate OSSN from benign entities and identify OSSN in the setting of co-existing ocular surface diseases [4, 10]. With a resolution of 2 to 7 μm, HR-OCT can confirm morphological evidence of disease.

When treating OSSN with medical therapy, most clinicians will provide topical therapy based on the clinical observation of tumor resolution. A current gap exists if clinical resolution indeed corresponds with complete tumor resolution. Incomplete treatment could potentially lead to recurrences. Thus, identifying and treating microscopic disease is crucial.

By providing an “optical biopsy,” HR-OCT can confirm disease presence and resolution. When the tumor appears clinically resolved to the physician, the HR-OCT can potentially identify the presence of residual disease. No study to date has evaluated the role of HR-OCT in detecting sub-clinical disease. The primary purpose of this study was to determine the frequency of sub-clinical OSSN detected by HR-OCT and to identify the timeline for complete HR-OCT resolution in those cases. The secondary goal was to identify possible predisposing risk factors for sub-clinical disease.

## Methods

### Study design

A retrospective chart review of 95 patients with OSSN identified through a pharmacy database at the Bascom Palmer Eye Institute from January 2013 to December 2018 was conducted. This retrospective study was approved by the institutional review board of the University of Miami and was compliant with the Health Insurance Portability and Accountability Act. Inclusion criteria included individuals with OSSN who were treated at the Bascom Palmer Eye Institute with topical chemotherapy as primary therapy and had resolution of their tumors. Patients who were considered non-responders to 5-FU or IFN were switched to an alternative agent**.** Exclusion criteria included chemotherapy use as an adjuvant to surgery, perilesional injection of chemotherapy, inadequate follow up, OSSN managed at an outside institution, and lack of concomitant HR-OCT images.

### Scanning protocol

All images were taken with the commercially available Optovue Avanti (Fremont, CA) and the Optovue RTvue (Fremont, CA) devices. Our protocol was to scan the entire tumor including additional 4 mm extra margins on initial presentation. Circumferential scans of the ocular surface were also performed at approximately every clock hour. Line scans were done manually by the technician with 1 to 3 mm of spacing in between the line scans. The initial pre-treatment tumor location was documented. Once the tumor had been treated, the documented area of the tumor location along with safety margins were rescanned.

### Disease definitions

**Fig. 1:**
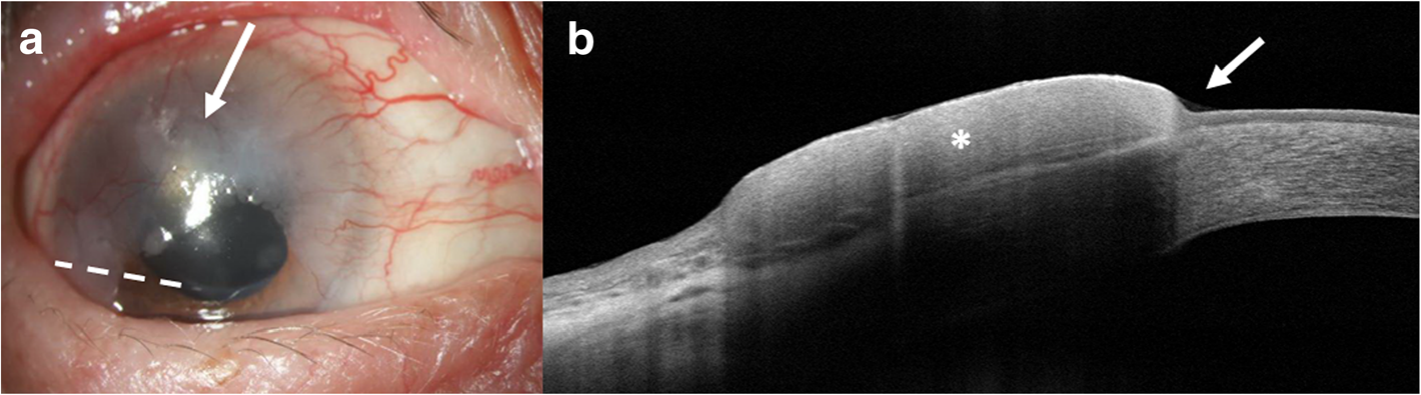
HR-OCT of OSSN. **a** A slit lamp photograph of the right eye of a superior flat/opalescent ocular surface squamous neoplasia (arrow). Dashed white line represents area of OCT scan. **b** High resolution optical coherence tomography (HR-OCT) reveals thickened and hyperreflective epithelium (*) with an abrupt transition point (arrow)

**Fig. 2:**
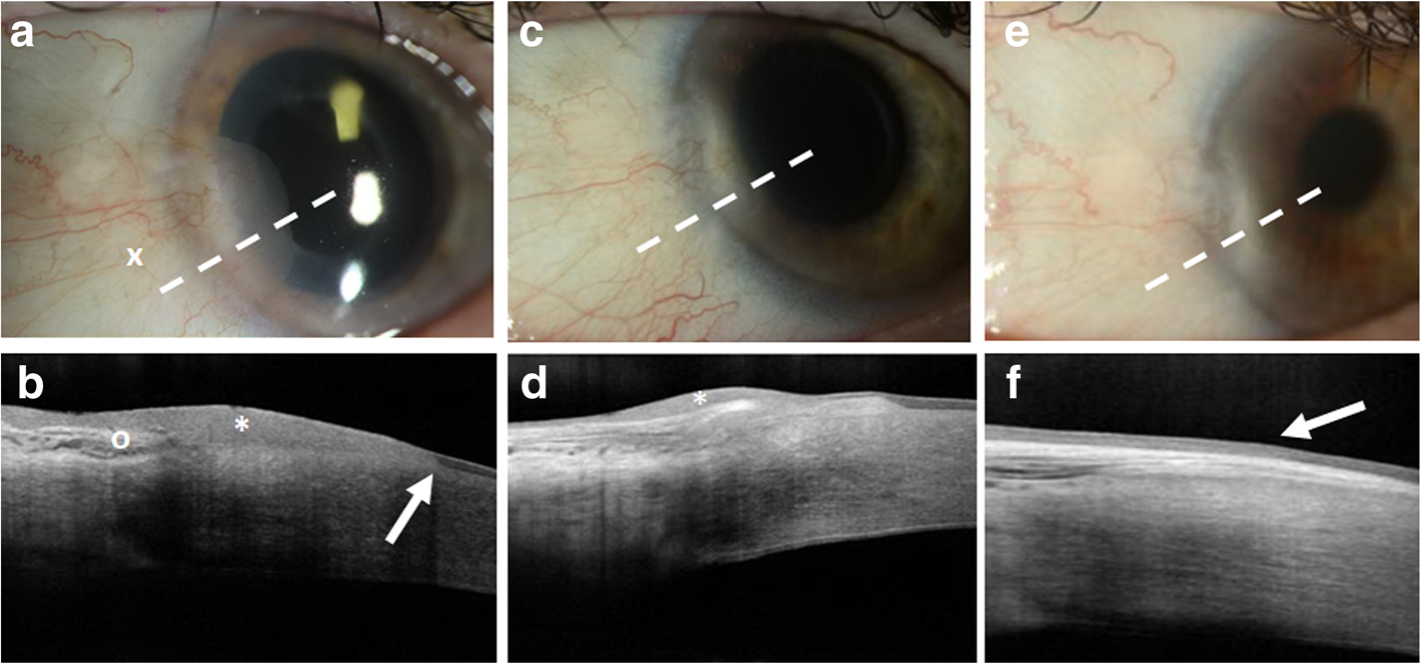
HR-OCT of sub-clinical OSSN. **a** Slit lamp image of the left eye of a flat/opalescent ocular surface squamous neoplasia (arrow) emanating from the head of a subtle pterygium (marked by an x). Note opalescent tissue on the cornea from 7 to 9 o’clock. **b** High resolution optical coherence tomography (HR-OCT) revealed thickened, hyperreflective, epithelium (marked by an *) with an abrupt transition (arrow). Directly below is a subepithelial “stringy” hyperreflectivity consistent with the pterygium (marked by an o). **c** After 4 cycles of 5-flourouracil (5-FU), the flat/opalescent lesion at the head of the pterygium was no longer clinically visible. **d** HR-OCT reveals improvement but persistent sub-clinical disease (marked by an *) with residual hyperreflective thickened epithelium. **e** After an additional 2 cycles of 5-FU, the lesion remains clinically resolved. **f** Now the HR OCT confirms normalized, thin epithelium (arrow). Sub-epithelial scarring consistent with the pterygium remains as expected

All patients diagnosed with OSSN had classic features identified clinically and by HR-OCT. These included thickened, hyperreflective epithelium, with an abrupt transition from normal to abnormal epithelium on HR-OCT (Fig. 1). Clinical resolution was defined as complete resolution by slit lamp examination of all OSSN features, which could include leukoplakia, gelatinous, papillomatous, flat opacity, and/or a nodule. Group 1 included patients with complete clinical resolution aligned with full HR-OCT normalization. Group 2 included patients with sub-clinical disease with the presence of OSSN features on HR-OCT but no evidence of clinical disease (hyperreflective epithelium, abrupt transition from normal to abnormal epithelium, Fig. 2).

Clinical response time was defined as the number of cycles of 5-FU or months of IFN needed to achieve clinical resolution from the start of treatment. In those with sub-clinical disease, additional treatment with cycles of 5-FU or months of IFN needed to achieve HR-OCT resolution after clinical resolution was documented. Recurrence was defined as a reappearance of a similar lesion in the same location after complete clinical resolution of the original tumor.

### Treatment protocol

Patients were treated with topical 5-FU 1% 4 times a day for 7 days with a 21-day drug holiday. Patients were treated with topical IFN at a concentration of 1 million International Units (IU)/mL 4 times a day without interruption. Both treatments were continued until clinical resolution was noted. When the clinical picture of resolution was confirmed by HR-OCT, the treatment was terminated. In cases of sub-clinical disease seen by HR-OCT, additional treatment was provided until normalization of HR-OCT was documented. In patients that did not respond to the original therapy, the patient was switched to a second chemotherapeutic agent.

### Demographic and tumor data

Demographic information extracted from the medical record included age, sex, race, ethnicity and OSSN risk factors (skin cancer, human papilloma virus, HIV, smoking and sun exposure, pterygium, prior pterygium surgery, history of OSSN). Tumor characteristics included the involved eye, location site, tumor size, involved ocular structures, unifocality or multifocality and appearance based on descriptions and photograph (leukoplakic, gelatinous, papillomatous, flat, nodular). Tumors were also staged based on the American Joint Committee on Cancer (AJCC) [11]. Treatment information included primary modality of treatment including dose, frequency and length of treatment and need for secondary treatment modalities.

### Study outcomes

Given that residual disease may lead to recurrences, the main outcome measure was the frequency of sub-clinical disease seen by HR-OCT when there was no evidence of clinical disease, and then the time needed on therapy to reach complete OCT resolution. Secondary outcomes included identification of risk factors for sub-clinical disease.

### Statistical analyses

Statistical analyses were conducted with the Statistical Package for the Social Sciences, SPSS 22.0 (SPSS Inc., Chicago, IL). Continuous variables were compared using independent Student’s *t*-test and categorical variables using the Chi squared analysis. Kaplan–Meier analysis were used to analyze recurrence rates. Univariable analyses were performed to determine which factors predicted sub-clinical disease. A multivariable analysis was then run to consider the additional contribution of demographic factors. Data was stored at the Bascom Palmer Eye Institute.

## Results

### Study population

A total of 95 patients (95 eyes) were identified whose OSSN was successfully treated with topical chemotherapy as a primary agent. Overall, the majority of patients were white males in their early seventies (Table 1). There were no differences in demographics or exposures between the two groups other than current smoking which was more common in Group 2 (*p* = 0.03), and a history of skin cancer which was more common in Group 1 (p = 0.03).

**Table 1:** Demographics and clinical information of patients with clinical and sub-clinical ocular surface squamous neoplasia treated with 5-flourouracil or interferon alfa 2b eye drops

Characteristics	OSSN without Sub-clinical Disease (*n* = 90)	OSSN with Sub-clinical Diseasem (*n* = 16)	*P* Value
Patient Information			
Age (years), mean (SD)	68 (13)	70.4 (13)	0.45
Gender, male sex, % (n)	48 (49)	75 (12)	0.42
Race, % (n)			0.77
Black	5 (4)	6 (1)	
White	58 (49)	75 (12)	
Hispanic ethnicity, % (n)	23 (20)	56 (9)	0.85
Current smoker, % (n)	9 (8)	0 (0)	0.03
Sun exposure, % (n)^a^	58 (29)	100 (11)	0.06
History of pterygium, % (n)	21 (19)	73 (11)	0.67
History of OSSN, % (n)	10 (9)	0 (0)	0.36
History of skin cancer, % (n)	20 (15)	54 (7)	0.03
History of HIV, % (n)	1 (1)	0 (0)	0.65
History of HPV, % (n)	11 (7)	10 (1)	0.81
Clinical Information			
Involve eye, right % (n)	42 (38)	62 (10)	0.53
Area (mm^2^), mean (SD)	38 (34)	34 (25)	0.65
Treated with 5-FU, % (n)	54 (43)	44 (7)	0.43
Treated with IFN, % (n)	45 (36)	56 (9)	
Treated with both IFN or 5-FU, % (n)	14 (13)	12 (2)	0.47
Location % (n)^b^			
Nasal	45 (41)	56 (9)	0.75
Temporal	43 (39)	56 (9)	0.62
Superior	11 (10)	19 (2)	0.52
Inferior	26 (23)	37 (6)	0.51
Corneal involvement, % (n)	67 (60)	81 (13)	0.65
Corneal & conjunctival involvement, % (n)	50 (45)	56 (9)	0.94
Clinical AJCC stage, % (n)			0.10
T1a	14 (13)	25 (4)	
T2a	18 (16)	13 (2)	
T2b	5 (4)	6 (1)	
T3a	51 (45)	56 (9)	
Multifocal tumor, % (n)	14 (13)	12 (2)	0.69
Appearance, % (n)^c^			
Leukoplakia	25 (22)	37 (6)	0.46
Papillomatous	18 (16)	19 (3)	0.87
Flat/Opalescent	35 (31)	44 (7)	0.77
Gelatinous	37 (33)	44 (7)	0.91

*AJCC*= American Joint Committee on Cancer [5], *SD*= standard deviation, *HIV*= human immunodeficiency virus, *HPV*= human papillomavirus, *OSSN*= ocular surface squamous neoplasia

^a^Sun exposure was defined as patient noting to spend a large quantity of time outdoors

^b^Tumors could involve more than 1 quadrant; for example, a tumor involving the superior and temporal bulbar conjunctiva would be placed in both the superior and temporal location category

^c^Tumors could have more than 1 descriptor for appearance

Data are n (%) unless otherwise indicated

In Group 1 with clinically resolved disease, 79 patients (79 eyes, 83% of all patients) had simultaneous clinical and HR-OCT resolution of their OSSN, 43 of whom were first treated with IFN and 36 of whom were first treated with 5-FU. Of these patients, 16 were subsequently switched to a different topical agent due to persistence of clinically apparent disease.

In Group 2 with sub-clinical disease, sixteen individuals (17% of all patients) had evidence of sub-clinical OSSN by HR-OCT after the clinically apparent disease had resolved. Nine patients were first treated with IFN and seven with 5-FU. Of these patients, 2 were subsequently switched from IFN to 5-FU due to persistence of clinically apparent disease. After treating these patients with 5-FU, there was clinical resolution of OSSN, but persistent sub-clinical OSSN detected by HR-OCT. Both patients had complete HR-OCT resolution with additional treatment of 5-FU. Resolution of OSSN in Group 2 was confirmed in all cases by HR-OCT normalization. No statistical difference was seen between Group 1 and Group 2 with the need of a second topical chemotherapeutic agent (*p* = 0.47).

### Tumor characteristics

OSSN was frequently found nasally and temporally and less frequently inferiorly and superiorly on the conjunctival surface. Most tumors also involved the cornea. There were no statistically significant differences in any tumor characteristics between the groups with and without sub-clinical disease (Table 1).

### Risk factors for sub-clinical disease

We performed univariable analyses to evaluate which factors predicted the presence of sub-clinical disease. Individuals with a history of skin cancer had a 3.7-fold increased risk of having sub-clinical disease compared to those without a history of skin cancer (*p* = 0.04, 95% confidence interval (CI) 1.06–12.58). None of the other factors were predictive of sub-clinical disease. When considering the contribution of demographics in a multivariable analysis, a history of skin cancer remained a significant predictor of sub-clinical disease.

### Treatment information

In the 79 patients in Group 1, 4.0 ± 1.5 cycles of 5-FU and 3.8 ± 1.5 months of IFN were given to achieve clinical and HR-OCT resolution. In the 16 individuals in Group 2 with sub-clinical disease, the mean time to clinical resolution was 3.6 ± 1.0 cycles for 5-FU and 4.0 ± 0.0 months for IFN. An additional 2.1 ± 0.8 cycles for 5-FU and 1.2 ± 0.4 months for IFN were needed to achieve HR-OCT resolution of OSSN.

Individuals in Group 1 were followed for 24.0 ± 20.1 months after clinical and HR-OCT resolution. Individuals in Group 2 were followed for 24.0 ± 17.9 months after HR-OCT OSSN resolution was documented. There was no significant difference in follow up time between the two groups.

**Fig. 3:**
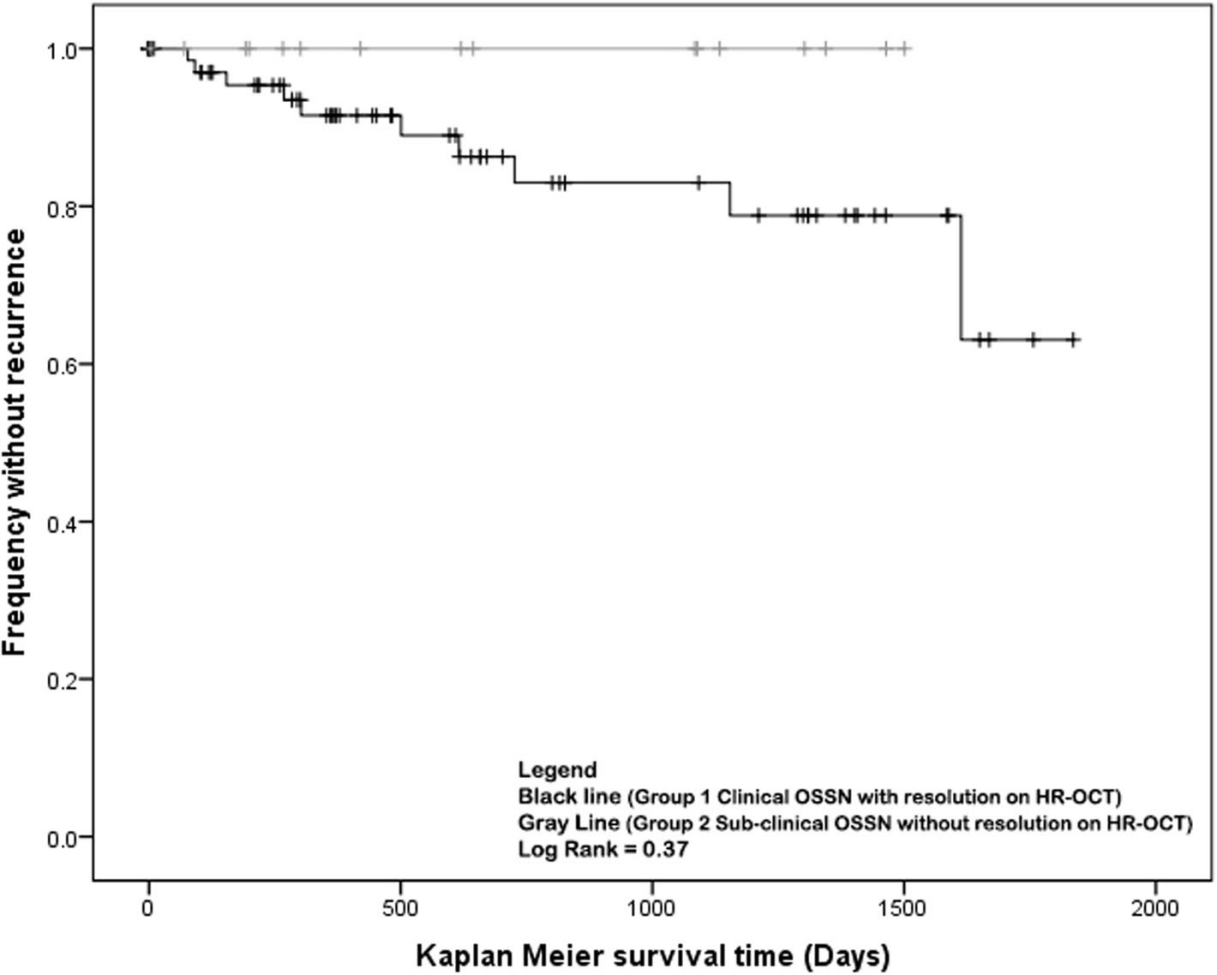
Kaplan-Meier Recurrence Time. Kaplan-Meier survival curve depicting time from clinical resolution to recurrence in the two groups

Recurrence in Group 1 were noted in 10 patients (12%) while no recurrences were noted in Group 2. Kaplan-Meier survival curves of OSSN recurrence showed a trend of increased recurrence in Group 1 with resolution clinically on HR-OCT compared to Group 2 with sub-clinical disease detected on HR-OCT (log rank = 0.37, Fig. 3). However, due to the limited number of recurrences, there was no statistically significant difference in time to recurrence.

## Discussion

This study found that sub-clinical OSSN was detected by HR-OCT in 17% of patients whose tumors were noted to have resolved clinically. While the clinical implications of sub-clinical disease are unknown, OSSN recurrences are noted in 0–28% of eyes treated with 5-FU and in 4–20% of eyes treated with IFN [12–28]. It can be postulated that recurrences may be driving residual disease or sub-clinical disease, especially since most occurred within the first year after stopping treatment. The goal of treatment is to completely eliminate all tumor cells and leave no microscopic residual disease.

The ability to visualize the tumor with the HR-OCT has the great benefit in helping to avoid premature termination of the chemotherapeutic intervention [3]. In addition, assuring tumor resolution with HR-OCT also can prevent over-use of topical chemotherapy which can lead to toxicity and additional unnecessary costs to the patient. Thus, HR-OCT optimizes the time on topical chemotherapy, potentially minimizing recurrence, toxicity and cost. The side effects of 5-FU included pain, redness, tearing, eyelid edema and keratopathy [7]. The side effects of IFN are minimal but can include mild conjunctival irritation [7]. The use of these agents has potential out of pocket costs as many insurance companies do not cover compounded medications.

In our study, patients in Group 2 with sub-clinical disease detected by HR-OCT were monitored vigilantly every 2 months and were treated with additional chemotherapy until the HR-OCT normalized. The recurrence rate was zero in this group. Once resolved, patients in both groups were followed every 4 months in the first year.

In contrast, patients in Group 1 with coinciding clinical and HR-OCT normalization had a trend toward higher recurrence rates. This may have been due to unknown tumor or patient factors but could also be explained by undetected sub-clinical disease. This emphasizes a limitation of our current HR-OCT technology, which does not automatically scan the entire ocular surface and thus may miss areas of sub-clinical disease. Currently, HR-OCT images are acquired manually by a technician with line scans that are spaced 1–3 mm apart. Areas of sub-clinical disease in regions between the line scans or in non-imaged areas may have been missed and resulted in the recurrences noted in the study.

We found that almost 20% of patients had sub-clinical disease detected by the HR-OCT. In those with detected sub-clinical disease, a mean of 2 additional cycles of 5-FU and 6 weeks of IFN were needed to achieve HR-OCT resolution. As such, when clinicians do not have access to HR-OCT, extending treatment by 2 additional cycles of 5-FU or 6 additional weeks of IFN treatment may be an appropriate buffer to treat possible sub-clinical disease.

As with all studies, our findings need to be considered in light of the study limitations which include its retrospective nature. Our identified frequency of 17% of cases having sub- clinical disease upon clinical resolution may be an underestimation. It is possible that areas of persistent disease were missed with current manual HR-OCT scanning protocols as mentioned above and some of the cases in Group 1 may have had undetected sub-clinical disease, and subsequent premature termination of topical chemotherapy. It is also possible that the longer treatment schedule independently affected the outcomes. Our study only evaluated patients under treatment regimens with 5-FU and IFN. Other treatment modalities may alter the rate of sub-clinical disease or recurrences. Unmeasured factors, such as tumor genetics and host immune response could underlie the differences seen in the groups. Finally, scanning with HR-OCT is currently done manually by a technician and the entire ocular surface is not automatically scanned. Hopefully, future software development will provide a technician-independent, automated imaging of the entire ocular surface.

## Conclusions

In conclusion, our study showed that 17% of patients had sub-clinical disease detected by HR-OCT technology. When topical chemotherapy was extended until resolution of the sub-clinical disease, the recurrence rate in this group was zero. The take home lesson from this data is that sub-clinical disease exists, and thus clinicians should consider treating patients with 2 additional cycles of 5-FU or 6 weeks of IFN past the point of clinical resolution. Future studies are needed to study whether this approach can improve disease outcomes.

This study adds to the potential role of HR-OCT in diagnosing and monitoring anterior segment conjunctival and corneal pathologies. We found that in some cases, the HR-OCT could identify residual sub-clinical disease in the setting of apparent clinical resolution and help guide the management of additional topical therapy.

## Data Availability

The datasets used and/or analyzed during the current study are available from the corresponding author upon reasonable request.
